# Comparative iron management in hemodialysis and peritoneal dialysis patients: a systematic review

**DOI:** 10.3389/fneph.2024.1488758

**Published:** 2024-11-27

**Authors:** Thomas S. van Lieshout, Anastasia K. Klerks, Osman Mahic, Robin W. M. Vernooij, Michele F. Eisenga, Brigit C. van Jaarsveld, Alferso C. Abrahams

**Affiliations:** ^1^ Department of Nephrology, Amsterdam UMC Location Vrije Universiteit Amsterdam, Research Institute Amsterdam Cardiovascular Sciences, Amsterdam, Netherlands; ^2^ Department of Internal Medicine, Northwest Clinics, Alkmaar, Netherlands; ^3^ Amsterdam Cardiovascular Sciences, Diabetes and Metabolism, Amsterdam, Netherlands; ^4^ Department of Nephrology and Hypertension, University Medical Center Utrecht, Utrecht, Netherlands; ^5^ Julius Center for Health Sciences and Primary Care, University Medical Center Utrecht, Utrecht University, Utrecht, Netherlands; ^6^ Division of Nephrology, Department of Internal Medicine, University of Groningen, University Medical Center Groningen, Groningen, Netherlands; ^7^ Nephrocare Diapriva Dialysis Center, Amsterdam, Netherlands

**Keywords:** iron therapy, anemia, hemodialysis, peritoneal dialysis, kidney failure

## Abstract

**Background:**

Patients with kidney failure undergoing dialysis often suffer from anemia. Iron deficiency, along with a shortage in erythropoietin, is a common cause. Peritoneal dialysis (PD) patients may have a different iron metabolism compared to hemodialysis (HD) patients. This study aims to compare both dialysis modalities regarding their differences in iron management.

**Methods:**

PubMed (MEDLINE) and Embase were screened for randomized controlled trials and observational studies including both patients on HD or PD with information on iron management. Outcomes for iron management for this systematic review included: prevalence of supplementation, route of administration, dose, frequency and hemoglobin and iron status parameters.

**Results:**

15 eligible studies (930,436 patients), of which 8 cohort and 7 cross-sectional, were analyzed. The prevalence of intravenous (IV) iron supplementation ranged from 11.7% to 84.4% in HD patients, compared to 1.6% to 49.0% in PD patients. Ten studies reported that HD patients only received IV iron, while five studies reported this for PD patients. For oral iron supplementation, three studies involved HD patients, whereas seven studies involved PD patients. The cumulative monthly IV iron dose ranged from 108 to 750 mg in the HD group, compared to 65 to 250 mg in the PD group. Hemoglobin levels ranged from 10.0 to 12.0 g/dL in HD patients, versus 9.6 to 11.9 g/dL in PD patients.

**Conclusion:**

Iron management differs between HD and PD patients, with HD patients receiving higher doses and more frequent IV iron. There was significant heterogeneity in the outcomes between the studies, primarily due to the lack of a uniform global policy on iron management. Despite these differences, hemoglobin levels and iron status parameters were comparable between the two groups. Future research should explore the underlying mechanisms and broader impacts of iron treatment, including patient-reported outcomes, to optimize anemia management and improve quality of life for dialysis patients.

**Systematic Review Registration:**

https://www.crd.york.ac.uk/prospero/, identifier CRD42022336970.

## 1 Introduction

Patients with chronic kidney disease (CKD) frequently suffer from anemia (1). With each stage, the prevalence of anemia increases, and is highest in patients with kidney failure with a prevalence of approximately 66% (2, 3). Anemia has a detrimental effect on a patient’s quality of life due to symptoms such as fatigue and weakness, and is also linked to increased mortality (1, 2). Several mechanisms contribute to anemia of CKD, the main pathway being a relative erythropoietin (EPO) deficiency due to an insufficient production in the damaged kidneys (4). The second most important contributor to anemia of CKD is iron deficiency, which includes two types: absolute iron deficiency and functional iron deficiency (5). The first is seen in patients with both low circulating iron concentrations and low or absent total body iron stores. It is due to a reduced iron intake, decreased iron absorption, gastrointestinal loss, and increased blood loss. Functional iron deficiency is seen when iron stores are adequate, but they cannot be utilized effectively for producing red blood cells (RBC) (5–7). It can result from chronic inflammation, also seen in other chronic diseases besides CKD, and the use of erythropoietin stimulating agents (ESA). ESA supplementation stimulates erythropoiesis, which can exhaust the existing amount of iron stored. Subsequently, this can lead to insufficient RBC production and EPO therapy resistance (5, 6). Both types of iron deficiencies are observed in dialysis patients, although some of the contributing factors are dependent on the type of dialysis modality (hemodialysis (HD) or peritoneal dialysis (PD)) (8, 9).

The KDIGO (Kidney Disease: Improving Global Outcomes) guidelines recommend the use of intravenous (IV) iron for HD patients given the significant body of evidence proving its advantage over oral formulations (7, 10, 11). Concerning the frequency and the amount of iron administered to HD patients, the large randomized controlled PIVOTAL trial has shown better results with a proactive, high-dose regimen as compared to a reactive, low-dose regimen (12, 13). However, there are still many questions unanswered regarding the optimal-dose finding and whether high IV doses (higher than in PIVOTAL) could be used, although observational data have shown that more intensive treatment strategies over a longer period of time are associated with an increased risk of mortality and infections (14–16).

It is believed that patients on PD generally have a different iron metabolism than HD patients and potentially require lower levels of iron supplementation (17). These patients do not suffer from the same frequent causes of iron loss as HD patients, such as significant blood loss because of the dialysis procedure (18, 19). Another difference may be the clinical characteristics between both modality groups, such as the lower level of inflammation and better residual kidney function (RKF) in PD patients. In addition, iron dosing and adjustment thereof could differ between HD and PD patients due to less frequent blood tests, and different policies on the dialysis modalities (18). However, increased demand due to ESA supplementation and reduced iron intake is also observed (20). The KDIGO guidelines recommend the use of IV iron in PD patients for the treatment of iron deficiency. However, there is markedly less literature on iron management in the PD population and previous guidelines have recommended oral iron supplementation instead of IV (10).

Previously, there was considerable interest in the differences between HD and PD patients, mostly expressed in terms of overall survival and quality of life years (21–24). Studies on iron management are, however, usually conducted within a specific single modality dialysis population, e.g., oral iron *vs*. IV iron in HD patients. Literature regarding differences in iron management between HD and PD is scarce. The majority of studies predominantly include one modality, mostly HD. This review aims to compile the available literature on the comparison of iron treatment in the management of anemia in HD and PD patients. We hypothesize that, in order to achieve comparable anemia and serum iron marker levels, the prevalence of iron supplementation is higher among HD patients, and that they more frequently receive IV iron, with higher doses and at increased frequency.

## 2 Methods

### 2.1 Search strategy and systematic review protocol

We conducted a systematic review following the PRISMA guidelines, the checklist is included in the supplementary material. The protocol with methods of the analysis and selection criteria were documented in advance in a protocol published on PROSPERO (no.: CRD42022336970), the protocol is listed in the supplementary material (58). The search was conducted using the PubMed and Embase databases in February 2024. The proposed search strategy was reviewed by an external clinical librarian and the final search included the following terms: hemodialysis, peritoneal dialysis, anemia, and iron (with all synonyms). The full details of the search strategy are listed in the supplementary material. Additional studies were identified by searching through reference lists and citations of the included studies.

### 2.2 Study design and population

After removing duplicates, two authors (AKK and OM) independently screened the titles and abstracts for eligibility. Final inclusion was based on consensus between both authors. Only randomized controlled trials and observational studies were deemed eligible for inclusion. Articles were selected if they included data on iron treatment in adult CKD patients (>18 years) receiving HD or PD. Iron outcome data included: prevalence of iron therapy, route of administration (IV of oral), iron therapy use, dose and frequency dose of administration, and anemia and iron status parameters. Articles that contained only one dialysis modality or articles that contained no outcomes of interest, were excluded. In addition, articles of which the full text was not available or the language was not in English were also excluded.

### 2.3 Data extraction and data analysis

Data extraction was performed by two authors (AKK and TSL) independently and again extraction was based on consensus. Due to the nature of this study, the presentation of the results will be done through a narrative synthesis. Given the expected heterogeneity and variation in outcome measures, a meta-analysis will not be conducted. The extracted data used for the study characteristics table included region, study design, sample size, mean age, sex and main study outcomes. The extracted data for the study outcome included data on prevalence of iron therapy, route of iron administration, mean iron dose, and frequency of iron administration, these data will be presented as a table of figure. Data on hemoglobin (Hb), ferritin and transferrin saturation (TSAT) values were also collected and presented in a figure. Data presented in a figure will also be presented as a table in the supplementary material.

### 2.4 Quality assessment

The Newcastle-Ottawa Scale (NOS) was used for methodological quality assessment (25). The risk of bias is based on three domains within the scale: selection (containing four items), comparability (containing one item) and outcome (containing three items). To assess the quality of included cohorts studies the NOS for cohort studies was used, the maximum number of stars rated in the assessment was nine. For cross-sectional studies an adapted NOS was used to optimize the risk of bias assessment, a maximum of ten stars could be scored (26). A study with a score of seven stars or higher was considered to be of high quality resulting in a low risk of bias, this applied to both scales (25). Examples of the used scales can be found in the supplementary material. Two authors (TSL and OM) independently evaluated the quality of the studies. Any discrepancies between the two authors on the quality was resolved through discussion.

## 3 Results

### 3.1 Literature search results

**Figure 1:**
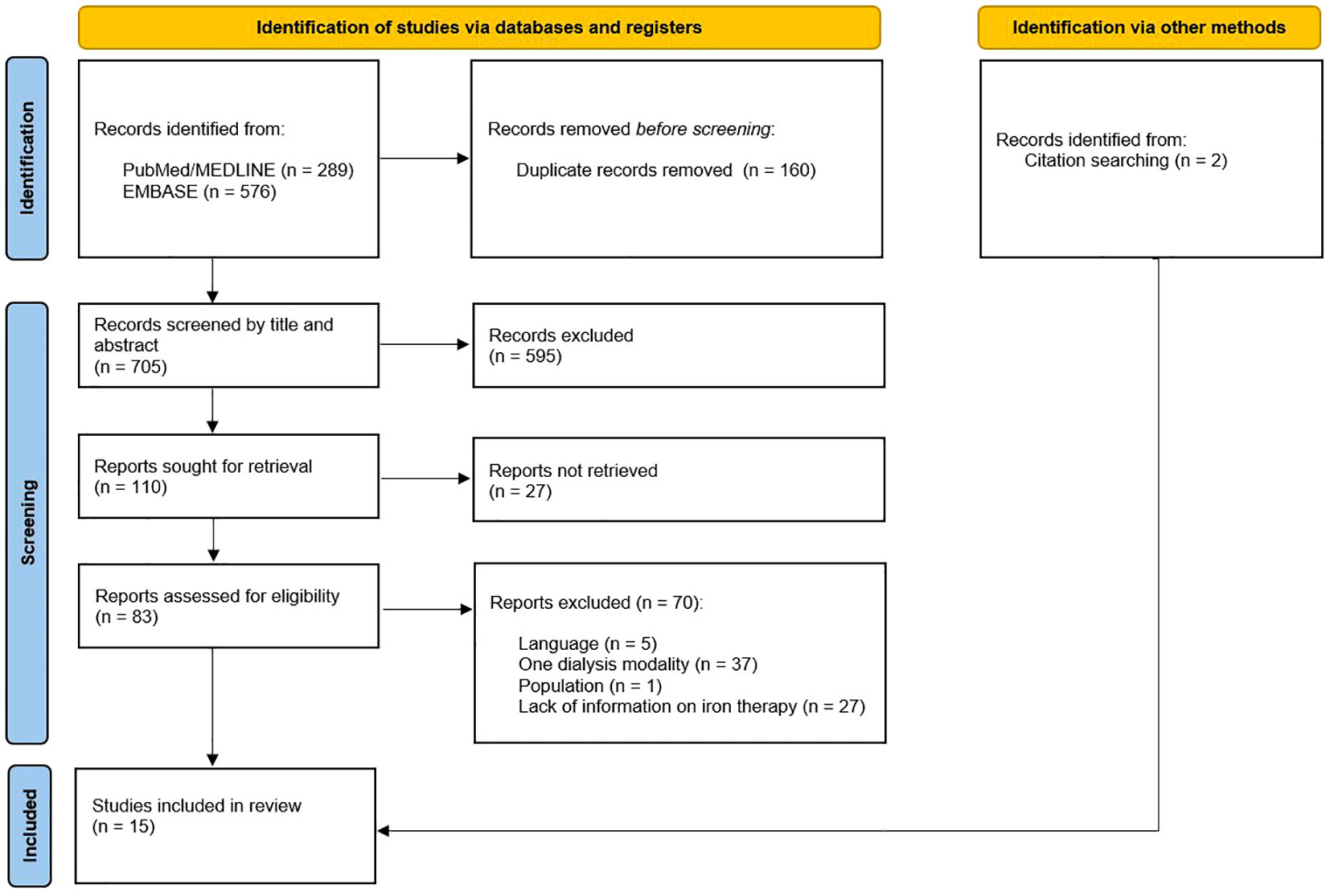
PRISMA flowchart (58).

After removal of duplicates, 705 articles were identified and screened by title and abstract using the pre-defined inclusion and exclusion criteria. The full text of the remaining 110 potentially relevant articles was then reviewed. After full-text screening, 97 articles were excluded due to the following reasons: language, one dialysis modality, population and no information on iron treatment. The remaining 13 articles were deemed relevant for this review. Lastly, another two articles were identified and included through citation screening. Thus, we included a total of 15 studies (**Figure 1**).

### 3.2 Study characteristics

The included studies are summarized in **Table 1**. Three were prospective cohort studies, five were retrospective cohort studies, and seven had a cross-sectional study design. Three studies were conducted in the United States of America, using the same Medicare registry in different time periods. St. Peter et al. included multiple cohorts in different time periods (1991, 1994, 2001 and 2002). To prevent overlap with patients of the other two studies only the 2002 cohort of St. Peter et al. was analyzed. The remaining studies were conducted in Europe (Austria, Poland, Spain, Sweden and Turkey), Asia (China, Japan and South Korea) and North America (Canada).

**Table 1:** Study characteristics.

Author, Year	Country	Study design	Total population		Mean age (SD) years		Sex, Male (%)		Main study outcomes
			HD (n)	PD (n)	HD (n)	PD (n)	HD (n)	PD (n)	
Bae, 2015 (27)	South Korea	Prospective cohort	1,594	876	58.0 (13.0)	54.0 (12.0)	56.7	56.8	ESA responsiveness on all-cause mortality
Chavers, 2004 (28)	USA	Retrospective cohort	352,291	3,9136	NA	NA	NA	NA	Prevalence of anemia
Coronel, 2003 (29)	Spain	Cross-sectional	69	63	65.0 (15.0)	56.0 (15.0)	58.0	51.0	ESA management by subcutaneous route, iron parameters, iPTH, ACEI and AIIA
Deger, 2013 (30)	Turkey	Cross-sectional	73	29	47.0 (16.0)	45.0 (15.0)	68.5	62.1	Serum levels of intact FGF23
Evans, 2020 (31)	Sweden	Retrospective cohort	2,337	708	Median [IQR], 70.0 [64.0; 80.0]	Median [IQR], 70.0 [64.0; 80.0]	67.0	67.0	Prevalence, management, and adverse clinical outcomes of renal anemia
Gao, 2023 (32)	China	Cross-sectional	70	50	60.6 (12.0)	56.9 (11.3)	74.2	54	Serum levels of hepcidin and reticulocyte hemoglobin equivalent
House, 1998 (33)	Canada	Retrospective cohort	157	126	57.2 (1.5)	57.5 (1.5)	59.2	57.1	Transfusion practices and rHuEpo use
Lim, 2019 (34)	South Korea	Prospective cohort	42	57	60.0 (9.5)	56.3 (9.3)	61.4	64.3	Serum levels of hepcidin
Malyszko, 2009 (35)	Poland	Cross-sectional	102	44	52.3 (12.5)	56.0 (15.0)	NA	NA	Serum levels of hepicidin and prohepcidin
Matsumura, 2020 (36)	Japan	Cross-sectional	55	14	Median [IQR], 71.0 [63.0; 79.0]	Median [IQR], 70.0 [60.0; 79.0]	60.0	50.0	Red blood cell age
Niikura, 2019 (37)	Japan	Cross-sectional	80	88	66.0 (12.0)	62.0 (14.0)	63.0	65.0	Serum levels of hepcidin-25 and serum levels of ferritin
St. Peter, 2005 (38)	USA	Retrospective cohort	241,770	13,491	NA	NA	52.7	52.7	IV iron use
Wetmore, 2015 (39)	USA	Retrospective cohort	256,942	17,842	NA	NA	NA	NA	ESA and IV iron use and dose, RBC transfusions and haemoglobin levels
Zhou, 2012 (40)	China	Cross-sectional	1,539	556	55.2 (15.3)	50.7 (15.0)	57.6	49.3	Serum levels of advanced oxidation protein products and prevalence of ischemic heart disease
Zitt, 2014 (41)	Austria	Prospective cohort	197	38	61.7 (13.7)	61.7 (13.7)	61.8	61.8	Iron supplementation and all-cause, cardiovascular- and sepsis-related mortality

HD, hemodialysis; PD, peritoneal dialysis; IV, intravenous; NA, Not available.

The total number of HD and PD patients was 857,318 and 73,118, respectively. The patient group sizes ranged from 42 to 352,291 in the HD group and 14 to 39,136 in the PD group. The mean age of the HD patients ranged from 47.0 to 66.0 years, and for PD patients from 45.0 to 62.0 years. Three studies provided no information on age for both the HD and PD patients (28, 38, 39). Three studies had iron management in HD and PD patients as one of its main outcomes (38, 39, 41). The remaining studies presented iron management either as baseline characteristics or as secondary outcome. Regarding the cohort studies the follow-up duration ranged from 6 months to 8 years.

### 3.3 Quality assessment

The quality assessment per study is presented in the supplementary material. The total scores of the cohort studies can be found in **Table 2** and the total scores of the cross-sectional studies in **Table 3**. Six of the eight included cohort studies were considered high quality. In the remaining cohort studies, there was a particularly low score on the comparability domain due to not controlling for confounding factors at all or leaving out important factor such as: age, sex, comorbidity, Hb and inflammation. Another reason for low scores on the outcome domain was due to inadequate follow-up. Four of the seven cross-sectional studies were of high quality. Studies with lower scores were primarily due to poor performance in the comparability domain, as they did not control for confounding factors.

**Table 2:** Newcastle-Ottawa Scale quality assessment of included cohort studies.

Author, Year	Selection	Comparability	Outcome	Total
	(max. 4 stars)	(max. 2 stars)	(max. 3 stars)	(max. 9 stars)
Bae, 2015 (27)	★★★★	★★	★★★	★★★★★★★★★
Chavers, 2004 (28)	★★★★	★★	★★	★★★★★★★★
Evans, 2020 (31)	★★★★	★★	★	★★★★★★★
House, 1998 (33)	★★★★	★★	★★★	★★★★★★★★★
Lim, 2019 (34)	★★★★		★★	★★★★★★
St. Peter, 2005 (38)	★★★★		★★	★★★★★★
Wetmore, 2015 (39)	★★★★	★★	★	★★★★★★★
Zitt, 2014 (41)	★★★★	★★	★★	★★★★★★★★

Stars are allotted within each domain based on the methodological quality of the study in that domain, a higher total number of stars indicates better overall methodological quality and lower risk of bias.

**Table 3:** Newcastle-Ottawa Scale quality assessment of included cross-sectional studies.

Author, Year	Selection	Comparability	Outcome	Total
	(max. 5 stars)	(max. 2 stars)	(max. 3 stars)	(max. 10 stars)
Coronel, 2003 (29)	★★★★		★★	★★★★★★
Deger, 2013 (30)	★★★★	★★	★★	★★★★★★★★
Gao, 2023 (32)	★★★★	★★	★★	★★★★★★★★
Malyszko, 2009 (35)	★★★★		★★	★★★★★★
Matsumura, 2020 (36)	★★★		★★	★★★★★
Niikura, 2019 (37)	★★★★	★	★★	★★★★★★★
Zhou, 2012 (40)	★★★★★		★★	★★★★★★★

Stars are allotted within each domain based on the methodological quality of the study in that domain, a higher total number of stars indicates better overall methodological quality and lower risk of bias.

### 3.4 Prevalence of iron therapy

**Figure 2:**
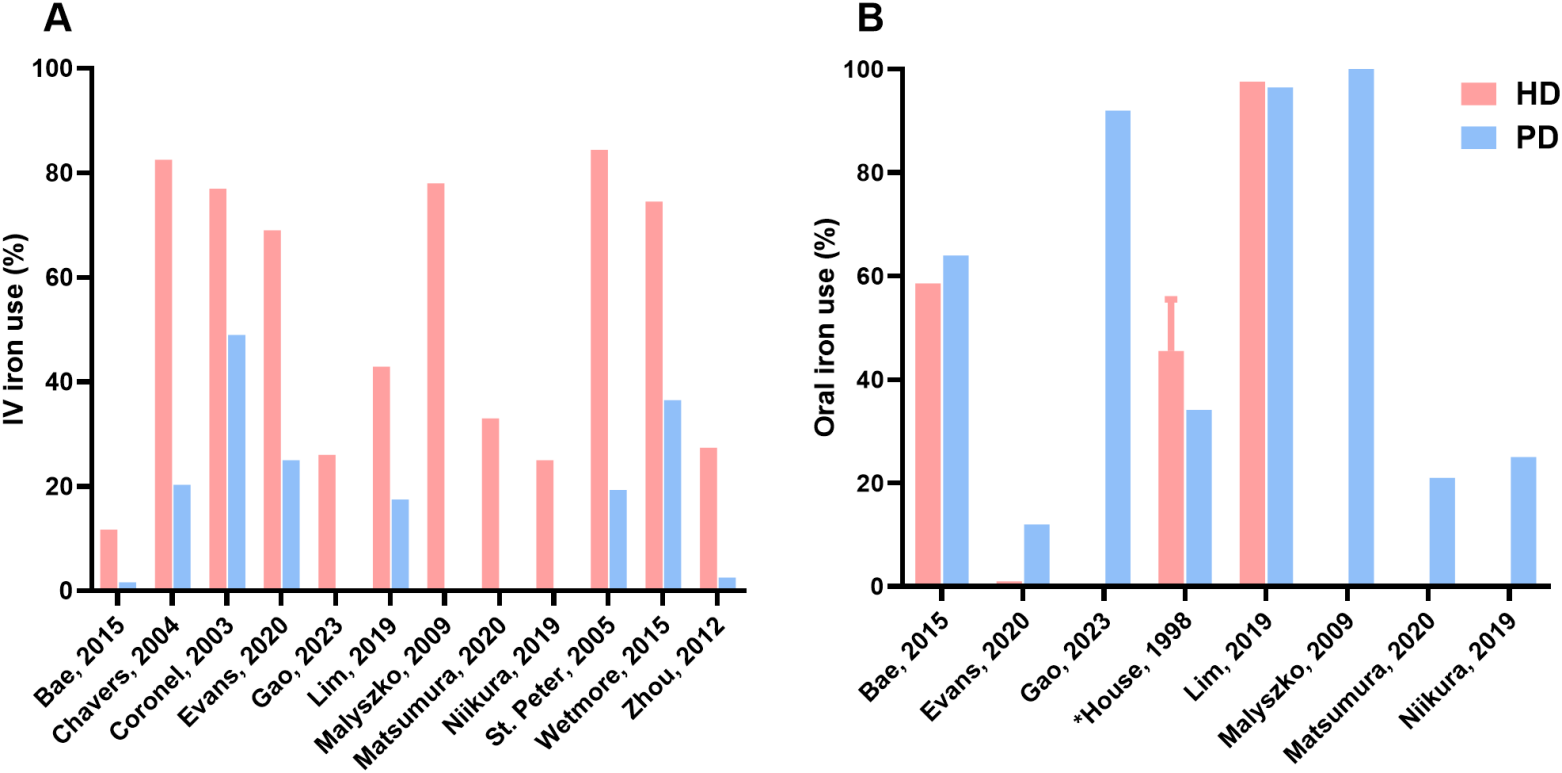
Prevalence of iron therapy. **(A)** Difference in IV iron use between HD and PD, **(B)** Difference in oral iron use between HD and PD; Gao, Malyszko, Matsumura, Niikura presented IV iron use prevalences for HD patients and oral iron use prevalences for PD patients; HD, hemodialysis; PD, peritoneal dialysis; IV, intravenous; *Value reported as range.

All studies included data on the prevalence of iron treatment (**Figure 2**). Overall, IV iron treatment percentages ranged from 11.7% to 84.4% in the HD group and 1.6% to 49.0% in the PD group. The three studies with the largest dialysis populations (based on the same registry with different time periods), showed the greatest difference in IV iron use between the dialysis modalities. First, Wetmore et al. reported a prevalence of 70.0% in HD to 19.7% in PD patients in their first quarter cohort of 2007, and a difference of 74.5% in HD to 36.5% in PD in the cohort of 2011 (39). Second, Chavers et al. reported 82.5% in HD to 20.3% in PD (28). Third, St. Peter et al. showed a prevalence of 84.4% in HD patients and 19.3% in PD patients (38).

Overall, oral iron treatment percentages ranged from 1% to 97.6% in the HD group and 12% to 100% in the PD group. Deger et al. made no distinction in IV and oral iron but showed a difference in iron therapy of 82.0% versus 72.0% in HD and PD patients, respectively (30). The three studies with dialysis patients receiving both IV and oral iron all showed differences between dialysis groups (27, 31, 34). Lim et al. had comparable numbers in regard to oral iron supplementation, i.e. 97.6% in HD patients and 96.5% in PD patients and a difference in IV iron supplementation, i.e. 42.9% in HD and 17.5% in PD (34). Evans et al. reported in HD patients 69% and 1% of IV iron and oral iron, respectively. In PD patients they reported 25% IV iron use and 12% oral iron (31). Bae et al. reported that 11.7% and 58.6% of HD patients received IV iron and oral iron, respectively. In PD patients, 1.6% received IV iron and 64% received oral iron (27). Niikura et al. showed similar percentages between both dialysis modalities: 25% IV iron in HD and 25% oral iron in PD (37).

### 3.5 Route of iron administration

All studies, except one, included data on the route of iron administration (**Table 4**). Three studies reported that both HD and PD patients received IV iron and oral iron (27, 31, 34). Five studies used only IV iron (28, 29, 38–40). In four studies HD patients received exclusively IV iron and PD patients only oral iron therapy (32, 35–37). No clear percentages on the route of iron administration were provided in three studies. Deger et al. made no distinction between IV and oral iron, and stated it merely as ‘iron therapy’ (30). House et al. reported the total iron use in the HD group (55.5%) but only stated that less than 10% was administered via the IV route, no further differences between oral and IV iron were available; for the PD group it was not explicitly stated whether iron use concerned IV iron or oral iron, however the oral route of administration was implied in the text (33). Zitt et al. stated that 94% of the total dialysis population received IV iron, there was no distinction between HD and PD (41).

**Table 4:** Route of iron administration.

Author, Year	Route of administration	
	HD	PD
Bae, 2015 (27)	IV & oral iron	IV & oral iron
Chavers, 2004 (28)	IV iron	IV iron
Coronel, 2003 (29)	IV iron	IV iron
Deger, 2013 (30)	*NA*	*NA*
Evans, 2020 (31)	IV & oral iron	IV & oral iron
Gao, 2023 (32)	IV iron	Oral iron
House, 1998 (33)	*NA*	*NA*
Lim, 2019 (34)	IV & oral iron	IV & oral iron
Malyszko, 2009 (35)	IV iron	Oral iron
Matsumura, 2020 (36)	IV iron	Oral iron
Niikura, 2019 (37)	IV iron	Oral iron
St. Peter, 2005 (38)	IV iron	IV iron
Wetmore, 2015 (39)	IV iron	IV iron
Zhou, 2012 (40)	IV iron	IV iron
Zitt, 2014 (41)*	94% of total used IV	94% of total used IV


HD, hemodialysis; PD, peritoneal dialysis; IV, intravenous; NA, Not available.

*No distinction was made between hemodialysis and peritoneal dialysis in reporting the route of administration.

### 3.6 Dose and frequency of iron therapy

**Figure 3:**
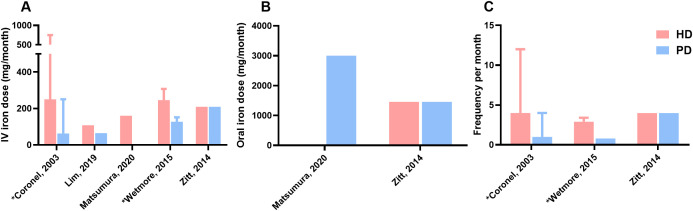
Dose and frequency of iron therapy **(A)**, Mean dose of IV iron administration per 30 days (month) in mg, **(B)**, Mean dose of oral iron administration per 30 days (month) in mg, **(C)**, Frequency of iron administration per 30 days (month); Matsutsumura presented IV iron dose for HD patients and oral iron dose for PD patients; Wetmore and Zitt described frequency only for patients receiving intravenous treatment; HD, hemodialysis; PD, peritoneal dialysis; Hb, hemoglobin; IV, intravenous; *Value reported as range.

As reported in **Figure 3**, information on the mean dose of iron administration was available in five studies. The mean dose of IV iron administered per 30 days in HD patients ranged from 108 to 750 mg/month and from 62.5 to 250 mg/month in PD patients. In the study by Lim et al. cumulative IV iron dose per treated patient was converted to mean dose/month. Detailed information on oral iron doses was not available in this study, only that they were similar between HD and PD patients (34). Oral iron administration was included in only two of the five studies. Matsumura et al. reported an oral iron dose of 3000 mg/month (100 mg/day) for PD patients, while Zitt et al. reported dosages of 1455 mg/month in both HD and PD patients (36, 41). Three studies reported the frequency of iron administration. Data were converted to iron administrations per 30 days. The frequency of IV iron administration in HD patients ranged from 2.9 to 12 times per 30 days. The frequency of IV iron administration ranged from 0.8 to 4 times per 30 days in PD patients.

### 3.7 Hemoglobin and iron status parameters

**Figure 4:**
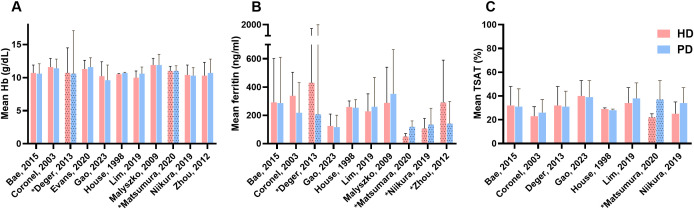
Anemia and iron serum markers. **(A)** Difference in mean Hb between HD and PD, **(B)** Difference in mean ferritin between HD and PD, **(C)** Difference between mean TSAT between HD and PD; HD, hemodialysis; PD, peritoneal dialysis; Hb, hemoglobin; TSAT, transferrin saturation; *Value reported as median with interquartile range.

**Figure 4** shows the hemoglobin values and the iron parameters ferritin and TSAT. The values presented in the table represent the entire HD or PD populations, including patients treated with iron and those who are not. Hb values in the HD patients ranged from 10.0 to 12.0 g/dL. In the PD patients the Hb values ranged from 9.6 to 11.9 g/dL Two studies reported statistically lower ferritin levels in HD patients compared to PD patients (35, 36), while three other studies reported higher ferritin levels in HD patients (29, 30, 40). Two studies reported lower percentages of TSAT in the HD group compared to the PD group (36, 37).

## 4 Discussion

### 4.1 Summary of findings

**Figure 5:**
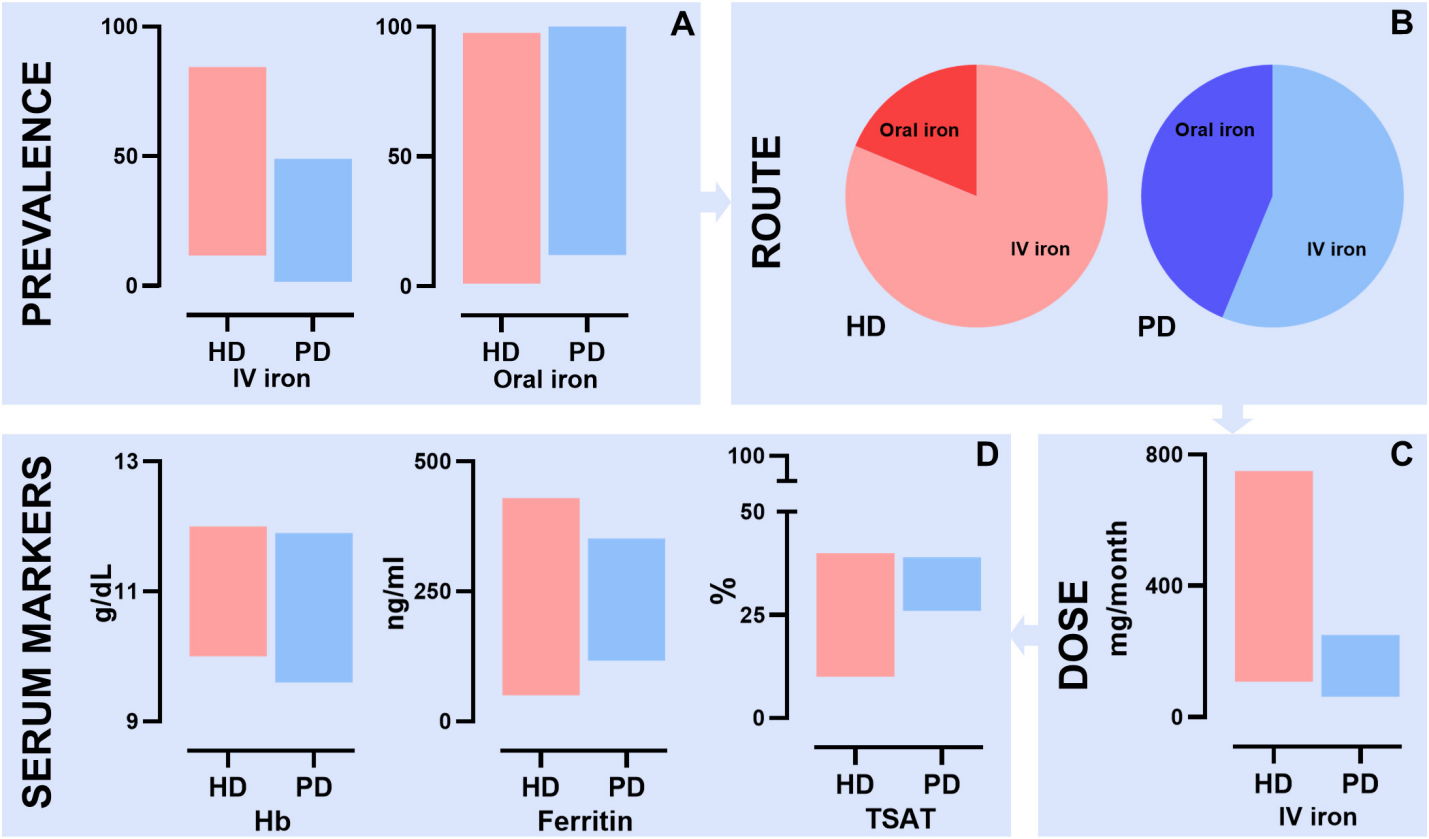
Summary of findings of the systematic review describing differences in iron management between HD and PD patients. **(A)** Prevalence of IV iron supplementation ranged from 11.7% to 84.4% in HD patients and 1.6% to 49.0% in PD patients. Prevalence of oral iron supplementation ranged from 1.0% to 97.6% in HD patient and 12.0% to 100.0% in PD patients; **(B)** Difference in route of iron administration between HD and PD patients; **(C)** Difference in mean IV iron dose in mg per month between HD and PD patients. HD patients ranged from 108 mg to 750 mg and PD patients from 62.5 mg to 250 mg; **(D)** Differences in anemia and iron serum markers between HD and PD patients. For Hb HD patients ranged from 10.0 to 12.0 g/dL and PD patients from 9.6 g/dL and 11.9 g/dL. For ferritin HD patients ranged from 50 ng/ml to 430 ng/ml and PD patient from 116.9 ng/ml to 352 ng/ml. For TSAT HD patients ranged from 10% to 40% and PD patient from 26% to 39%; HD, hemodialysis; PD, peritoneal dialysis; IV, intravenous; TSAT, transferrin saturation.

This systematic review aimed to summarize the available literature on the comparison of iron treatment in the management of anemia in HD and PD patients (see **Figure 5** for the summary of findings). The results of this review showed a heterogeneity in iron management between HD patients and PD patients across the included studies. A higher percentage of HD patients receives iron supplementation compared to PD patients. Also, HD patients predominantly receive IV iron, whereas PD patients receive either oral or IV iron. Moreover, the cumulative monthly IV dose of iron is higher in HD patients than in PD patients. Despite these differences in treatment, the hemoglobin and iron status parameters were largely comparable between the two groups. These findings suggest a difference in iron metabolism between the groups.

### 4.2 Prevalence of iron therapy

The results showed that the overall prevalence of iron use was higher in HD patients than in PD patients, supporting clinical observations. This difference in iron treatment was seen in all studies, but most noticeably in the studies by Wetmore et al., St. Peter et al., Chavers et al. and Evans et al. (28, 31, 38, 39). In their time-trend analysis from 2007 to 2011, Wetmore et al. concluded that IV iron use was consistently higher in HD patients (39). The causes of these differences between HD and PD patients are not explained clearly in the study itself, as not all factors for prescribing decisions and inherent differences between both dialysis groups could be identified due to the observational nature of their study (39). However, this finding is supported by previous research. PD patients are thought to require less iron supplementation, as they have a different iron metabolism and responsiveness to iron (10, 17). These patients experience less inflammation, generally have a slower decline of their residual kidney function, and do not suffer from the same blood loss related to the HD treatment (17, 19, 42). Furthermore, PD patients require substantially less ESA supplementation and lower ESA doses than HD patients, as was shown in a large cohort study of 139,103 HD and 10,527 PD patients in the United States (43). RBC production is stimulated by the use of ESAs and this can create what is called iron-restricted erythropoiesis: while total body iron stores are normal, the release of available iron to the bone marrow is insufficient and cannot keep up with this increased demand for erythropoiesis (44). As PD patients use less ESA, there is a decreased risk of relative iron depletion and therefore there could be less need for iron supplementation. This is further supported by the results of Wetmore et al.: PD patients consistently had a lower ESA use and ESA dose (39). Interestingly enough, during the 4 years of their study, IV iron use increased in both dialysis groups. However, IV iron use percentages increased more in PD patients than in HD patients (from 19,7% to 36.5% and from 70% to 74,5%, respectively). This increase in IV iron use in the PD population could be explained by possible changing perspectives on iron use concerning lower fears of IV iron safety, easier IV iron delivery, and oral iron tolerability issues (10, 31, 45, 46). Wetmore et al. also found a greater decrease in ESA use in PD patients compared to HD patients and this might also be related to the simultaneous increase in iron use (39). Previous studies, including the study conducted by Evans et al., on iron therapy prevalence reported low overall iron use in dialysis patients (31, 47). It is unclear whether this reported increase in IV iron use in PD patients in the study by Wetmore et al. is a rising trend, thus more up-to-date studies are needed to confirm this finding.

### 4.3 Route of administration

Second, the results showed that HD patients were more likely to be treated with IV iron than oral iron. This is in line with the current KDIGO anemia guidelines, which recommend that all dialysis patients with iron deficiency be treated with IV iron, and is further supported by the KDOQI, The NICE, and the ERBP guidelines (10, 48, 49). Many studies, including large RCTs, have shown IV iron to be more effective than oral iron in correcting Hb levels, increasing ferritin levels, and lowering ESA dosages in HD patients (11, 50). Furthermore, IV iron can be easily administered during HD treatment sessions as patients already visit a center multiple times a week and IV access is readily available. There have been several studies showing the same results in PD patients (51, 52), however, comparative research is still lacking in PD population (6). The KDIGO guidelines recommend PD patients be treated with IV iron instead of oral iron, as the evidence is considered to be of sufficient quality (10).

Some of the included studies in this review still reported patients on oral iron therapy. More often in PD patients, but several studies reported similar prevalence in HD patients. Most surprising was the prospective cohort study by Lim et al. that included an oral iron prescription of almost 100% in both HD and PD patients, while also administering IV iron in both groups, albeit more in HD patients (34). The study describes in its methodology that each dialysis patient begins with oral iron supplementation, which is supplemented with IV iron if the patient does not achieve the target Hb level. This approach seems consistent with national policies during that time, as reflected in a 2017 Korean national cohort study that outlines a similar step-up strategy (47). Several factors could explain the use of oral iron here: the severity of the iron deficiency, IV iron intolerance, a patients’ own choice, financial reasons, preservation of venous access sites, and easier self-administration at home (10, 49, 53, 54). The latter two factors are especially relevant for PD patients.

Based on this review, it is unclear whether general practice differs from current guidelines and research recommendations because of different hospital and/or country policies, different patient’s needs, or if it is simply a finding in this specific selection of studies (18). None of the studies in this review reported the general rationale behind the iron prescription and it is therefore important for future studies to include this data. This will result in a better overview of the current iron management practices.

### 4.4 Dose and frequency

Aside from the prevalence of iron use, the results also showed that the mean dose of IV iron and frequency of administration was higher in HD patients compared to PD patients. This could once again reflect the higher need for iron in the HD group. Coronel et al. and Wetmore et al. both showed that HD patients received approximately double the amount of IV iron per 30 days (29, 39). This indicates a more high-dose IV iron regimen in the HD group, instead of a maintenance iron regimen. The PIVOTAL trial showed that patients on HD that received a high-dose IV iron regimen proactively (median monthly dose of 264 mg) required lower doses of ESA’s administered and that this approach was superior in terms of significantly lower risk of death or major nonfatal cardiovascular as compared to lower doses of IV iron reactively (median monthly dose of 145 mg) (13). However, Wetmore et al. also noted that even though from 2007 to 2011 the frequency of administrations had increased, the actual doses supplemented decreased (39). This could indicate a move towards a maintenance iron approach instead of a reactive approach.

The doses in PD patients were lower in all studies, which could be a sign of less need, but also of caution in administering high doses of IV iron due to presumed higher risks of infections, adverse cardiovascular events, and higher risk of death. The PIVOTAL trial reported no association with any of these outcomes, however, when comparing higher doses of IV iron with lower doses (13). Besides a higher frequency of iron administration, Wetmore et al. also reported a more variable frequency in HD patients (39). These patients visit the hospital or dialysis clinic several times a week, while PD patients receive treatment at home and only visit the hospital for checkups. Markers of iron status can be monitored more frequently in HD patients and adjusted as necessary, while iron levels in PD patients are typically only monitored every few months (49). However, only a few studies reported the doses and frequency of iron administration, making the generalization of these findings difficult.

### 4.5 Hemoglobin levels and iron status parameters

Lastly, serum ferritin and TSAT levels are frequently used to assess iron status. A recent meta-analysis conducted by Wang et al. on the effect of HD and PD on renal anemia, included a total of 14 studies and showed no significant differences between HD and PD patients for levels of ferritin, TSAT and Hb (55). However, this meta-analysis did not include data on iron therapy differences between dialysis groups. The findings in our review are comparable to their meta-analysis, as no large differences can be seen between dialysis modalities. However, in all studies included in this review that reported Hb, ferritin and TSAT levels, numbers were given for the entire dialysis group regardless of whether patients received iron treatment or not. And as most studies had a varying number of HD or PD patients on iron treatment, it is difficult to distinguish the effect of iron therapy on ferritin and TSAT levels between HD and PD patients. The current KDIGO guidelines (2012) recommend iron supplementation for all adult CKD dialysis patients if TSAT is below 30% and ferritin is below 500 ng/ml or if an increase in Hb concentration or a decrease in ESA dose is desired (10). Most dialysis patients in the included studies published after 2012 (release of the KDIGO guidelines) had ferritin levels below the recommended 500 ng/ml, but TSAT > 30%. In that regard, no large differences can be seen between patients on HD and PD. However, the two studies that reported the overall lowest iron use in HD patients, showed a median TSAT <30%. Niikura et al. reported TSAT values of 25% in HD patients and Matsumura et al. a median TSAT of 22% in HD patients (36, 37). Both studies originate from Japan, where the national guidelines recommend maintaining lower ferritin levels (below 300 µg/L), which consequently leads to lower transferrin saturation (TSAT) percentages (56). Furthermore, it is unlikely that these results hold much significance, as both studies included only a small number of patients and had a high risk of bias.

Regarding Hb levels, no significant differences between dialysis modalities could be seen in all studies. The three largest studies included in this review did not include either Hb, ferritin or TSAT parameters in their study (28, 38, 39). Analyzing the other studies, mostly with smaller patient populations and high or unclear risk of bias, would not provide useful information for this review. Furthermore, these values are difficult to compare between studies due to different timepoints of measurement (before versus after an intervention or median over time). Other research aimed at these specific outcomes would be more valuable.

### 4.6 Strengths and limitations

The strength of this systematic review lies in its rigorous methodology, *a priori* described in a protocol, including an extensive search strategy including studies over the whole world. The aim of this review was to provide a general overview of the differences in iron treatment between patients receiving HD and PD. Studies conducted among both dialysis populations would be most representative to answer this research question. However, this review identified a definite lack of such studies conducted among both dialysis populations and assessing iron treatment. It is therefore necessary for future research to include larger sample sizes of both HD and PD patients, a study design with low risk of bias, adjustments for confounders and preferably a prospective cohort design with sufficient follow-up. The results of such studies would be beneficial to clinical practice, as iron therapy plays an essential part in the treatment of dialysis patients.

This study has several limitations. Firstly, there was a large heterogeneity among all studies in patient characteristics, methodology and available data. For example, important factors that could influence iron therapy, such as dialysis duration and ESA use differed greatly between studies. Wetmore et al. only reported IV iron use in patients receiving ESA supplementation, while most other studies included non-ESA users as well (39). Other important factors possibly related to iron therapy, such as transfusion and bleeding events, were also excluded by some studies. Furthermore, most studies were conducted in different countries, with different policies on iron treatment as shown in the PDOPPS study (18). Due to this inconsistency and heterogeneity among studies, no statistical analyses were performed, and no statistical significance could be attributed to the results. Secondly, the majority of patients included in this review were acquired from three studies. These retrospective studies all used the same Medicare registry from the United States, albeit in different time periods (28, 38, 39). Thirdly, this review did not distinguish between the different types of HD (conventional, daily and nocturnal) and PD (continues ambulatory PD and automated PD) due to limited number of included studies. And lastly, this review did not include information on possible risks related to iron therapy such as the safety of serum ferritin and TSAT upper limits, iron overload, risk of infection and oxidative stress (45, 57). While this is relevant for the topic, it goes beyond the scope of this study.

## 5 Conclusions

In conclusion, iron management is markedly different between HD and PD patients. Not only the route of administration was different, with HD patients being more likely to receive IV, but also the prevalence, administered iron doses and frequencies were all higher in HD patients compared to PD patients. Serum markers were comparable between the two modalities. These findings suggest different types of iron metabolism exist between the modalities, but future studies are needed to further investigate a possible underlying mechanism. Additionally, it is also important to investigate the relationship between iron and iron treatment on other than biochemical outcomes. Certainly, anemia management in dialysis patient should also include aspects like patient-reported outcomes, in order to develop an optimal strategy for improving quality of life of our dialysis patients.

## Data Availability

The original contributions presented in the study are included in the article/**Supplementary Material**. Further inquiries can be directed to the corresponding author.

## References

[1] Stauffer ME Fan T . Prevalence of anemia in chronic kidney disease in the United States. PloS One. (2014) 9:e84943–e. doi: 10.1371/journal.pone.0084943 PMC3879360 24392162

[2] Eschbach JW Adamson JW . Anemia of end-stage renal disease (ESRD). Kidney Int. (1985) 28:1–5. doi: 10.1038/ki.1985.109 3900528

[3] Kovesdy CP Davis JR Duling I Little DJ . Prevalence of anaemia in adults with chronic kidney disease in a representative sample of the United States population: analysis of the 1999-2018 National Health and Nutrition Examination Survey. Clin Kidney J. (2023) 16:303–11. doi: 10.1093/ckj/sfac240 PMC9900579 36755833

[4] Gunnell J Yeun JY Depner TA Kaysen GA . Acute-phase response predicts erythropoietin resistance in hemodialysis and peritoneal dialysis patients. Am J Kidney diseases: Off J Natl Kidney Foundation. (1999) 33:63–72. doi: 10.1016/S0272-6386(99)70259-3 9915269

[5] Batchelor EK Kapitsinou P Pergola PE Kovesdy CP Jalal DI . Iron deficiency in chronic kidney disease: updates on pathophysiology, diagnosis, and treatment. J Am Soc Nephrology: JASN. (2020) 31:456–68. doi: 10.1681/ASN.2019020213 PMC7062209 32041774

[6] Gafter-Gvili A Schechter A Rozen-Zvi B . Iron deficiency anemia in chronic kidney disease. Acta haematologica. (2019) 142:44–50. doi: 10.1159/000496492 30970355

[7] Gutiérrez OM . Treatment of iron deficiency anemia in CKD and end-stage kidney disease. Kidney Int Rep. (2021) 6:2261–9. doi: 10.1016/j.ekir.2021.05.020 PMC8418942 34514189

[8] Hayat A Haria D Salifu MO . Erythropoietin stimulating agents in the management of anemia of chronic kidney disease. Patient preference adherence. (2008) 2:195–200. doi: 10.2147/ppa.s2356 19920963 PMC2769266

[9] Hörl WH . Iron therapy for renal anemia: how much needed, how much harmful? Pediatr Nephrol (Berlin Germany). (2007) 22:480–9. doi: 10.1007/s00467-006-0405-y PMC1805051 17206511

[10] McMurray JJV Parfrey PS Adamson JW Aljama P Berns JS Bohlius J . Kidney disease: Improving global outcomes (KDIGO) anemia work group. KDIGO Clin Pract guideline anemia chronic Kidney disease. Kidney Int Supplements. (2012) 2:279–335. doi: 10.1038/kisup.2012.37

[11] Shepshelovich D Rozen-Zvi B Avni T Gafter U Gafter-Gvili A . Intravenous versus oral iron supplementation for the treatment of anemia in CKD: an updated systematic review and meta-analysis. Am J Kidney Diseases. (2016) 68:677–90. doi: 10.1053/j.ajkd.2016.04.018 27321965

[12] Babitt JL Eisenga MF Haase VH Kshirsagar AV Levin A Locatelli F . Controversies in optimal anemia management: conclusions from a Kidney Disease: Improving Global Outcomes (KDIGO) Conference. Kidney Int. (2021) 99:1280–95. doi: 10.1016/j.kint.2021.03.020 33839163

[13] Macdougall IC White C Anker SD Bhandari S Farrington K Kalra PA . Intravenous iron in patients undergoing maintenance hemodialysis. New Engl J Med. (2018) 380:447–58. doi: 10.1056/NEJMoa1810742 30365356

[14] Liu F . Proactive high-dose IV iron is preferred therapy in ESKD patients: COMMENTARY. Kidney360. (2022) 3:214–6. doi: 10.34067/KID.0004892021 PMC8967635 35378018

[15] Li X Cole SR Kshirsagar AV Fine JP Stürmer T Brookhart MA . Safety of dynamic intravenous iron administration strategies in hemodialysis patients. Clin J Am Soc Nephrol. (2019) 14:728–37. doi: 10.2215/CJN.03970318 PMC6500950 30988164

[16] Bailie GR Larkina M Goodkin DA Li Y Pisoni RL Bieber B . Data from the Dialysis Outcomes and Practice Patterns Study validate an association between high intravenous iron doses and mortality. Kidney Int. (2015) 87:162–8. doi: 10.1038/ki.2014.275 25075769

[17] Rostoker G . When should iron supplementation in dialysis patients be avoided, minimized or withdrawn? Semin Dial. (2019) 32:22–9. doi: 10.1111/sdi.12732 PMC7379289 29956370

[18] Perlman RL Zhao J Fuller DS Bieber B Li Y Pisoni RL . International anemia prevalence and management in peritoneal dialysis patients. Perit Dial Int. (2019) 39:539–46. doi: 10.3747/pdi.2018.00249 31582465

[19] van Eck van der Sluijs A Abrahams AC Rookmaaker MB Verhaar MC Bos WJW Blankestijn PJ . Bleeding risk of haemodialysis and peritoneal dialysis patients. Nephrol Dialysis Transplantation. (2021) 36:170–5. doi: 10.1093/ndt/gfaa216 PMC7771974 33130878

[20] Nissenson AR Strobos JUR . Iron deficiency in patients with renal failure. Kidney Int. (1999) 55:S18–21. doi: 10.1046/j.1523-1755.1999.055Suppl.69018.x 10084282

[21] Atapour A Nasr S Boroujeni AM Taheri D Dolatkhah S . A comparison of the quality of life of the patients undergoing hemodialysis versus peritoneal dialysis and its correlation to the quality of dialysis. Saudi J Kidney Dis transplantation: an Off Publ Saudi Center Organ Transplantation Saudi Arabia. (2016) 27:270–80. doi: 10.4103/1319-2442.178259 26997380

[22] Lee C-C Sun C-Y Wu M-S . Long-term modality-related mortality analysis in incident dialysis patients. Peritoneal Dialysis international: J Int Soc Peritoneal Dialysis. (2009) 29:182–90. doi: 10.1177/089686080902900213 19293356

[23] McDonald SP Marshall MR Johnson DW Polkinghorne KR . Relationship between dialysis modality and mortality. J Am Soc Nephrology: JASN. (2009) 20:155–63. doi: 10.1681/ASN.2007111188 PMC2615722 19092128

[24] van de Luijtgaarden MWM Jager KJ Segelmark M Pascual J Collart F Hemke AC . Trends in dialysis modality choice and related patient survival in the ERA-EDTA Registry over a 20-year period. Nephrology dialysis transplantation: Off Publ Eur Dialysis Transplant Assoc - Eur Renal Assoc. (2016) 31:120–8. doi: 10.1093/ndt/gfv295 26311215

[25] Wells GA Shea B O’Connell D Peterson J Welch V Losos M . The Newcastle-Ottawa Scale (NOS) for assessing the quality of nonrandomised studies in meta-analyses. (2000). Available online at: https://www.ohri.ca/programs/clinical_epidemiology/oxford.asp.

[26] Herzog R Álvarez-Pasquin MJ Díaz C Del Barrio JL Estrada JM Gil Á. Are healthcare workers’ intentions to vaccinate related to their knowledge, beliefs and attitudes? A systematic review. BMC Public Health. (2013) 13:154. doi: 10.1186/1471-2458-13-154 23421987 PMC3602084

[27] Bae MN Kim SH Kim YO Jin DC Song HC Choi EJ . Association of erythropoietin-stimulating agent responsiveness with mortality in hemodialysis and peritoneal dialysis patients. PloS One. (2015) 10:e0143348–e. doi: 10.1371/journal.pone.0143348 PMC4654568 26588085

[28] Chavers BM Roberts TL Herzog CA Collins AJ St. Peter WL . Prevalence of anemia in erythropoietin-treated pediatric as compared to adult chronic dialysis patients. Kidney Int. (2004) 65:266–73. doi: 10.1111/j.1523-1755.2004.00357.x 14675059

[29] Coronel F Herrero J Montenegro J Pérez C Gandara A Conesa J . Erythropoietin requirements: A comparative multicenter study between peritoneal dialysis and hemodialysis. J nephrology. (2003) 16:697–702. 14733416

[30] Deger SM Erten Y Pasaoglu OT Derici UB Reis KA Onec K . The effects of iron on FGF23-mediated Ca–P metabolism in CKD patients. Clin Exp Nephrology. (2013) 17:416–23. doi: 10.1007/s10157-012-0725-0 23180041

[31] Evans M Bower H Cockburn E Jacobson SH Barany P Carrero J-J . Contemporary management of anaemia, erythropoietin resistance and cardiovascular risk in patients with advanced chronic kidney disease: a nationwide analysis. Clin Kidney J. (2020) 13:821–7. doi: 10.1093/ckj/sfaa054 PMC7577763 33123358

[32] Gao Z Hu Y Gao Y Ma X Hu Z . The association of hepcidin, reticulocyte hemoglobin equivalent and anemia-related indicators on anemia in chronic kidney disease. Medicine. (2023) 102:17. doi: 10.1097/MD.0000000000033558 PMC10145874 37115087

[33] House AA Pham B Pagé DE . Transfusion and recombinant human erythropoietin requirements differ between dialysis modalities. Nephrol Dialysis Transplantation. (1998) 13:1763–9. doi: 10.1093/ndt/13.7.1763 9681725

[34] Lim J-H Park Y Lee S Do J-Y Kim SH Han S . Association of hepcidin with anemia parameters in incident dialysis patients: differences between dialysis modalities. Ther Apheresis Dialysis. (2019) 24:4–16. doi: 10.1111/1744-9987.12837 31090188

[35] Malyszko J Malyszko JS Kozminski P Mysliwiec M . Type of renal replacement therapy and residual renal function may affect prohepcidin and hepcidin. Renal Failure. (2009) 31:876–83. doi: 10.3109/08860220903216071 20030521

[36] Matsumura K Okumiya T Sugiura T Takahashi N Yamamoto Y Kikuchi S . Shortened red blood cell age in patients with end-stage renal disease who were receiving haemodialysis: a cross-sectional study. BMC Nephrology. (2020) 21:418–. doi: 10.1186/s12882-020-02078-z PMC7526359 32993543

[37] Niikura T Maruyama Y Nakashima S Matsuo N Tanno Y Ohkido I . Hepcidin/ferritin ratios differ among non-dialyzed chronic kidney disease patients, and patients on hemodialysis and peritoneal dialysis. Ther apheresis dialysis: Off peer-reviewed J Int Soc Apheresis Japanese Soc Apheresis Japanese Soc Dialysis Ther. (2019) 23:341–6. doi: 10.1111/1744-9987.12773 30411489

[38] St. Peter WL Obrador GT Roberts TL Collins AJ . Trends in intravenous iron use among dialysis patients in the United States (1994-2002). Am J Kidney Diseases. (2005) 46:650–60. doi: 10.1053/j.ajkd.2005.06.018 16183420

[39] Wetmore JB Peng Y Monda KL Kats AM Kim DH Bradbury BD . Trends in anemia management practices in patients receiving hemodialysis and peritoneal dialysis: A retrospective cohort analysis. Am J Nephrol. (2015) 41:354–61. doi: 10.1159/000431335 26107376

[40] Zhou Q Wu S Jiang J Tian J Chen J Yu X . Accumulation of circulating advanced oxidation protein products is an independent risk factor for ischaemic heart disease in maintenance haemodialysis patients. Nephrology. (2012) 17:642–9. doi: 10.1111/j.1440-1797.2012.01640.x 22738256

[41] Zitt E Sturm G Kronenberg F Neyer U Knoll F Lhotta K . Iron supplementation and mortality in incident dialysis patients: an observational study. PloS One. (2014) 9:e114144. doi: 10.1371/journal.pone.0114144 25462819 PMC4252084

[42] Marrón B Remón C Pérez-Fontán M Quirós P Ortíz A . Benefits of preserving residual renal function in peritoneal dialysis. Kidney Int. (2008) 73:S42–51. doi: 10.1038/sj.ki.5002600 18379546

[43] Duong U Kalantar-Zadeh K Molnar MZ Zaritsky JJ Teitelbaum I Kovesdy CP . Mortality associated with dose response of erythropoiesis-stimulating agents in hemodialysis versus peritoneal dialysis patients. Am J nephrology. (2012) 35:198–208. doi: 10.1159/000335685 PMC3326284 22286821

[44] Wish JB . Assessing iron status: beyond serum ferritin and transferrin saturation. Clin J Am Soc Nephrol. (2006) 1:S4 LP–S8. doi: 10.2215/CJN.01490506 17699374

[45] Hougen I Collister D Bourrier M Ferguson T Hochheim L Komenda P . Safety of intravenous iron in dialysis: A systematic review and meta-analysis. Clin J Am Soc Nephrol. (2018) 13:457–67. doi: 10.2215/CJN.05390517 PMC5967668 29463597

[46] Coyne DW Kapoian T Suki W Singh AK Moran JE Dahl NV . Ferric gluconate is highly efficacious in anemic hemodialysis patients with high serum ferritin and low transferrin saturation: results of the dialysis patients’ Response to IV iron with elevated ferritin (DRIVE) study. J Am Soc Nephrol. (2007) 18:975. doi: 10.1681/ASN.2006091034 17267740

[47] Ryu SR Park SK Jung JY Kim YH Oh YK Yoo TH . The prevalence and management of anemia in chronic kidney disease patients: result from the koreaN cohort study for outcomes in patients with chronic kidney disease (KNOW-CKD). J Korean Med science. (2017) 32:249–56. doi: 10.3346/jkms.2017.32.2.249 PMC5219990 28049235

[48] Locatelli F Bárány P Covic A De Francisco A Del Vecchio L Goldsmith D . Kidney Disease: Improving Global Outcomes guidelines on anaemia management in chronic kidney disease: a European Renal Best Practice position statement. Nephrol Dialysis Transplantation. (2013) 28:1346–59. doi: 10.1093/ndt/gft033 23585588

[49] National Clinical Guideline C . Anaemia management in chronic kidney disease: update 2021. In: NICE guideline [NG203]. National Institue for Health and Care Excellence (2021). Available online at: https://www.nice.org.uk.

[50] Macdougall IC Tucker B Thompson J Tomson CR Baker LR Raine AE . A randomized controlled study of iron supplementation in patients treated with erythropoietin. Kidney Int. (1996) 50:1694–9. doi: 10.1038/ki.1996.487 8914038

[51] Ahsan N . Intravenous infusion of total dose iron is superior to oral iron in treatment of anemia in peritoneal dialysis patients: a single center comparative study. J Am Soc Nephrology: JASN. (1998) 9:664–8. doi: 10.1681/ASN.V94664 9555669

[52] Johnson D Herzig K Gissane R Campbell S Hawley C Isbel N . Oral versus intravenous iron supplementation in peritoneal dialysis patients. Peritoneal Dialysis Int. (2001) 21:S231–S5. doi: 10.1177/089686080102103S41 11887827

[53] Vanholder R Davenport A Hannedouche T Kooman J Kribben A Lameire N . Reimbursement of dialysis: a comparison of seven countries. J Am Soc Nephrol. (2012) 23:1291–8. doi: 10.1681/ASN.2011111094 22677554

[54] Rastogi A Lerma EV . Anemia management for home dialysis including the new US public policy initiative. Kidney Int Supplements. (2021) 11:59–69. doi: 10.1016/j.kisu.2020.12.005 PMC7983021 33777496

[55] Wang W-N Zhang W-L Sun T Ma F-Z Su S Xu Z-G . Effect of peritoneal dialysis versus hemodialysis on renal anemia in renal in end-stage disease patients: a meta-analysis. Renal failure. (2017) 39:59–66. doi: 10.1080/0886022X.2016.1244079 27852131 PMC6014401

[56] Yamamoto H Nishi S Tomo T Masakane I Saito K Nangaku M . Japanese society for dialysis therapy: guidelines for renal anemia in chronic kidney disease. Renal Replacement Ther. (2015) 3:36. doi: 10.1186/s41100-017-0114-y

[57] Del Vecchio L Longhi S Locatelli F . Safety concerns about intravenous iron therapy in patients with chronic kidney disease. Clin Kidney J. (2016) 9:260–7. doi: 10.1093/ckj/sfv142 PMC4792617 26985378

[58] Page MJ McKenzie JE Bossuyt PM Boutron I Hoffmann TC Mulrow CD . The PRISMA 2020 statement: an updated guideline for reporting systematic reviews. BMJ. (2021) 372:n71. doi: 10.1136/bmj.n71 33782057 PMC8005924

