# Supplementary material for: Comparative iron management in hemodialysis and peritoneal dialysis patients: a systematic review
Source: Front Nephrol. 2024 Nov 27;4:1488758. doi: 10.3389/fneph.2024.1488758 (PMC11631840; doi:10.3389/fneph.2024.1488758)
Supplement: Supplementary file 1 [file DataSheet1.pdf]

Supplementary Material

Table of content

Supplementary Material 1

1. PRISMA 2020 Checklist 2
2. PROSPERO protocol 5
3. Search strategy 10
4. Newcastle-Ottawa Scales for cohort and cross-sectional studies 11
5. Quality assessment per study using the Newcastle-Ottawa Scale 15
6. Supplementary table prevalence of iron therapy 45
7. Supplementary table dose and frequency of iron therapy 46
8. Supplementary table anemia and iron serum markers 47

1. PRISMA 2020 Checklist

Section and Topic	Item #	Checklist item	Location where item is reported
**TITLE**			
Title	1	Identify the report as a systematic review.	P1
**ABSTRACT**			
Abstract	2	See the PRISMA 2020 for Abstracts checklist.	P2
**INTRODUCTION**			
Rationale	3	Describe the rationale for the review in the context of existing knowledge.	P3-4
Objectives	4	Provide an explicit statement of the objective(s) or question(s) the review addresses.	P4
**METHODS**			
Eligibility criteria	5	Specify the inclusion and exclusion criteria for the review and how studies were grouped for the syntheses.	P5
Information sources	6	Specify all databases, registers, websites, organisations, reference lists and other sources searched or consulted to identify studies. Specify the date when each source was last searched or consulted.	P5
Search strategy	7	Present the full search strategies for all databases, registers and websites, including any filters and limits used.	SUPP-P10
Selection process	8	Specify the methods used to decide whether a study met the inclusion criteria of the review, including how many reviewers screened each record and each report retrieved, whether they worked independently, and if applicable, details of automation tools used in the process.	P5
Data collection process	9	Specify the methods used to collect data from reports, including how many reviewers collected data from each report, whether they worked independently, any processes for obtaining or confirming data from study investigators, and if applicable, details of automation tools used in the process.	P5
Data items	10a	List and define all outcomes for which data were sought. Specify whether all results that were compatible with each outcome domain in each study were sought (e.g. for all measures, time points, analyses), and if not, the methods used to decide which results to collect.	P5
	10b	List and define all other variables for which data were sought (e.g. participant and intervention characteristics, funding sources). Describe any assumptions made about any missing or unclear information.	P5
Study risk of bias assessment	11	Specify the methods used to assess risk of bias in the included studies, including details of the tool(s) used, how many reviewers assessed each study and whether they worked independently, and if applicable, details of automation tools used in the process.	P6
Effect measures	12	Specify for each outcome the effect measure(s) (e.g. risk ratio, mean difference) used in the synthesis or presentation of results.	NA
Synthesis methods	13a	Describe the processes used to decide which studies were eligible for each synthesis (e.g. tabulating the study intervention characteristics and comparing against the planned groups for each synthesis (item #5)).	NA
	13b	Describe any methods required to prepare the data for presentation or synthesis, such as handling of missing summary statistics, or data conversions.	NA

Section and Topic	Item #	Checklist item	Location where item is reported
	13c	Describe any methods used to tabulate or visually display results of individual studies and syntheses.	P5
	13d	Describe any methods used to synthesize results and provide a rationale for the choice(s). If meta-analysis was performed, describe the model(s), method(s) to identify the presence and extent of statistical heterogeneity, and software package(s) used.	NA
	13e	Describe any methods used to explore possible causes of heterogeneity among study results (e.g. subgroup analysis, meta-regression).	NA
	13f	Describe any sensitivity analyses conducted to assess robustness of the synthesized results.	NA
Reporting bias assessment	14	Describe any methods used to assess risk of bias due to missing results in a synthesis (arising from reporting biases).	P6
Certainty assessment	15	Describe any methods used to assess certainty (or confidence) in the body of evidence for an outcome.	NA
**RESULTS**			
Study selection	16a	Describe the results of the search and selection process, from the number of records identified in the search to the number of studies included in the review, ideally using a flow diagram.	P7
	16b	Cite studies that might appear to meet the inclusion criteria, but which were excluded, and explain why they were excluded.	NA
Study characteristics	17	Cite each included study and present its characteristics.	P7
Risk of bias in studies	18	Present assessments of risk of bias for each included study.	P7-8 & SUPP-P15-44
Results of individual studies	19	For all outcomes, present, for each study: (a) summary statistics for each group (where appropriate) and (b) an effect estimate and its precision (e.g. confidence/credible interval), ideally using structured tables or plots.	P8-10
Results of syntheses	20a	For each synthesis, briefly summarise the characteristics and risk of bias among contributing studies.	NA
	20b	Present results of all statistical syntheses conducted. If meta-analysis was done, present for each the summary estimate and its precision (e.g. confidence/credible interval) and measures of statistical heterogeneity. If comparing groups, describe the direction of the effect.	NA
	20c	Present results of all investigations of possible causes of heterogeneity among study results.	NA
	20d	Present results of all sensitivity analyses conducted to assess the robustness of the synthesized results.	NA
Reporting biases	21	Present assessments of risk of bias due to missing results (arising from reporting biases) for each synthesis assessed.	NA
Certainty of evidence	22	Present assessments of certainty (or confidence) in the body of evidence for each outcome assessed.	NA
**DISCUSSION**			
Discussion	23a	Provide a general interpretation of the results in the context of other evidence.	P11-15

Section and Topic	Item #	Checklist item	Location where item is reported
	23b	Discuss any limitations of the evidence included in the review.	P15-16
	23c	Discuss any limitations of the review processes used.	P15-16
	23d	Discuss implications of the results for practice, policy, and future research.	P16
**OTHER INFORMATION**			
Registration and protocol	24a	Provide registration information for the review, including register name and registration number, or state that the review was not registered.	P5 & SUPP-P5-9
	24b	Indicate where the review protocol can be accessed, or state that a protocol was not prepared.	P5
	24c	Describe and explain any amendments to information provided at registration or in the protocol.	SUPP-P5-9
Support	25	Describe sources of financial or non-financial support for the review, and the role of the funders or sponsors in the review.	P17
Competing interests	26	Declare any competing interests of review authors.	P17
Availability of data, code and other materials	27	Report which of the following are publicly available and where they can be found: template data collection forms; data extracted from included studies; data used for all analyses; analytic code; any other materials used in the review.	NA

2. PROSPERO protocol

NIHR | National Institute for Health Research

PROSPERO
International prospective register of systematic reviews

Differences in iron management between patients receiving hemodialysis and peritoneal dialysis: a systematic review

To enable PROSPERO to focus on COVID-19 submissions, this registration record has undergone basic automated checks for eligibility and is published exactly as submitted. PROSPERO has never provided peer review, and usual checking by the PROSPERO team does not endorse content. Therefore, automatically published records should be treated as any other PROSPERO registration. Further detail is provided here.

Citation

T.S. van Lieshout, A.K. Klerks, O Mahic, R.W.M. Vernooij, M.F. Eisenga, B.C. van Jaarsveld, A.C. Abrahams. Differences in iron management between patients receiving hemodialysis and peritoneal dialysis: a systematic review.PROSPERO 2022 CRD42022336970 Available from: https://www.crd.york.ac.uk/prospero/display_record.php?ID=CRD42022336970

Review question

What is the difference in iron management between patients receiving hemodialysis and peritoneal dialysis regarding theprevalence of iron treatment, the route of administration, the dosage and the frequency?

What is the difference in relevant laboratory outcomes to iron treatment between patients receiving hemodialysis andperitoneal dialysis?

Searches

A systematic search will be conducted using the MEDLINE, EMBASE and The Cochrane Library databases. The proposed search strategy was reviewed by an external clinical librarian. The attached document shows the full details ofthe search strategy. Additional studies were identified by searching through reference lists and citations of the includedstudies.

Types of study to be included

Only clinical trials and observational studies will be included.

Condition or domain being studied

The domain of this systematic review will be anemia treatment in patients undergoing different dialysis modalities,specifically focusing on iron management.

Participants/population

Articles will be selected if they included data on iron treatment in adult end-stage kidney disease patients (>18 years) receiving hemodialysis or peritoneal dialysis. Iron treatment includes: dose of administration, frequency of treatment,route of iron supplementation (intravenous or oral), and type of iron supplement.

Articles that contain only one dialysis modality or articles that contain no outcomes of interest, will be excluded.

Intervention(s), exposure(s)

The main intervention will be hemodialysis.

Comparator(s)/control

The main control will be peritoneal dialysis.

Context

Only articles in English will be included. Articles of which the full text is not available will be excluded.

Main outcome(s)

Primarily, the study outcome will include data on the prevalence of iron use, the route of administration, the iron dose and the frequency.

The prevalence of iron use will be defined as the percentage of dialysis patient undergoing either oral or intravenous treatment. The iron dose is defined as the cumulative dose of iron in mg per 30 days. The frequency is defined as the cumulative frequency per 30 days.

This review aims to compile the available information on the comparison between the management of anamia in HD and PD patients, specifically with a focus on iron supplementation.

Additional outcome(s)

Secondary outcomes will include relevant laboratory outcomes to iron management like: hemoglobin, ferritin and transferrin saturation (TSAT).

Data extraction (selection and coding)

Two reviewers will independently screen the titles and abstracts after the search for eligibility. Potentially relevant articles will be assessed according to pre-defined the inclusion and exclusion criteria. Any disagreement will be resolved by discussion until consensus is reached or by consulting a third author. Endnote (v20.1) will be used for the selection and recording of the included articles.

Data extraction will be performed by two reviewers and will be checked by a third reviewer. Again, any disagreement will be resolved by discussion between the three reviewers. If consensus could not be reached between the three reviewers a fourth reviewer will be consulted. The extracted data that will be used for the study characteristics table will include: research methodology, region, sample size, mean age, and main study outcomes. The extracted data that will be used for the study outcome table will include data on iron use, route of iron administration, mean iron dose, and frequency of iron administration. Data on hemoglobin, ferritin and TSAT values will also be collected and will be presented in a table. Extracted data will be recorded in Windows Excel (Microsoft Office 2016).

Risk of bias (quality) assessment

Risk of bias will be determined for the outcome of iron treatment using the Risk of Bias Assessment Tool for Non-randomized Studies (RoBANS). The following six domains will be assessed: selection of participants, confounding variables, measurement of exposure, blinding of outcome assessments, incomplete outcome data, and selective outcome reporting. Articles will be scored as high risk of bias, low risk of bias or unclear. The assessment will be performed by two reviewers independently. In case of uncertainty, a third reviewer will be consulted.

Results will be visualized using the web app robvis (www.riskofbias.info). The overall risk of bias for a study will be calculated using three key domains: selection of participants, confounding variables, and incomplete outcome data. A study will be categorized as having high, low, or unclear risk of bias when more than one key domain has the same assessment. If all three key domains have different assessments, the overall risk of bias will be categorized as unclear.

Strategy for data synthesis

Studies considered acceptable for inclusion will be subjected to descriptive data synthesis. Different tables will included to summarize the data, if a p-value is given in the study this will be presented as well.

Analysis of subgroups or subsets

Not applicable

Contact details for further information

T.S. van Lieshout

t.s.vanlieshout@amsterdamumc.nl

Organisational affiliation of the review

Department of Nephrology, Amsterdam UMC location Vrije Universiteit Amsterdam, Amsterdam, the Netherlands

Department of Nephrology and Hypertension, University Medical Center Utrecht, Utrecht, the Netherlands

Department of Internal Medicine, Northwest Clinics, Alkmaar, the Netherlands

Department of Internal Medicine, Division of Nephrology, University Medical Center Groningen, Groningen, the Netherlands

Review team members and their organisational affiliations [1 change]

T.S. van Lieshout. Department of Nephrology, Amsterdam UMC location Vrije Universiteit Amsterdam, Research institute Amsterdam Cardiovascular Sciences, the Netherlands

A.K. Klerks. Department of Nephrology and Hypertension, University Medical Center Utrecht, Utrecht, the Netherlands.

O Mahic. Department of Nephrology and Hypertension, University Medical Center Utrecht, Utrecht, the Netherlands.

Dr R.W.M. Vernooij. Julius Center for Health Sciences and Primary Care, University Medical Center Utrecht, Utrecht University, Utrecht, The Netherlands

Dr M.F. Eisenga. Division of Nephrology, Department of Internal Medicine, University of Groningen, University Medical Center Groningen, Groningen, The Netherlands

Dr B.C. van Jaarsveld. Department of Nephrology, Amsterdam UMC location Vrije Universiteit Amsterdam, Research institute Amsterdam Cardiovascular Sciences, the Netherlands

Dr A.C. Abrahams. Department of Nephrology and Hypertension, University Medical Center Utrecht, Utrecht, the Netherlands.

Type and method of review

Systematic review

Anticipated or actual start date [1 change]

01 January 2024

Anticipated completion date [1 change]

01 July 2024

Funding sources/sponsors

Department of Nephrology, Amsterdam UMC location Vrije Universiteit Amsterdam, Amsterdam, the Netherlands

Department of Nephrology and Hypertension, University Medical Center Utrecht, Utrecht, the Netherlands

Conflicts of interest

Language

English

Country

Netherlands

Stage of review [1 change]

Review Completed not published

Subject index terms status

Subject indexing assigned by CRD

Subject index terms

Humans; Iron; Peritoneal Dialysis; Renal Dialysis

Date of registration in PROSPERO

12 June 2022

Date of first submission

01 June 2022

Stage of review at time of this submission [1 change]

Stage	Started	Completed
Preliminary searches	Yes	Yes
Piloting of the study selection process	Yes	Yes
Formal screening of search results against eligibility criteria	Yes	Yes
Data extraction	Yes	Yes
Risk of bias (quality) assessment	Yes	Yes
Data analysis	Yes	Yes

Revision note

Start date changed into actual start date and completion date added. Contributing authors added.

The record owner confirms that the information they have supplied for this submission is accurate and complete and they understand that deliberate provision of inaccurate information or omission of data may be construed as scientific misconduct.

The record owner confirms that they will update the status of the review when it is completed and will add publication details in due course.

Versions

12 June 2022

12 June 2022

11 July 2024

3. Search strategy

Database	Search
PubMed	("Peritoneal Dialysis"[Mesh] OR "Hemodialysis, Home"[Mesh] OR (("Renal Dialysis"[Mesh:noexp] OR "Kidneys, Artificial"[Mesh] OR hemodialys*[tiab] OR haemodialys*[tiab] OR hemo-dialys*[tiab] OR haemo-dialys*[tiab] OR renal dialys*[tiab] OR dialysis modalit*[tiab] OR artificial kidney*[tiab]) AND (home[tiab] OR homebased[tiab])) OR peritoneal dialys*[tiab] OR peritoneum dialys*[tiab])<br>AND<br>((iron[MeSH Terms]) OR (ferrous sulfate[Title/Abstract])) OR (ferrous gluconate[Title/Abstract])) OR (losferron[Title/Abstract])) OR (ferrous fumarate[Title/Abstract])) OR (ferrous chloride[Title/Abstract])) OR (iron dextran[Title/Abstract])) OR (ferric gluconate[Title/Abstract])) OR (iron sucrose[Title/Abstract])) OR (Ferric saccharate[Title/Abstract])) OR (iron saccharate[Title/Abstract])) OR (venofer[Title/Abstract])) OR (ferric carboxymaltose[Title/Abstract])) OR (Ferinject[Title/Abstract])) OR (Ferric derisomaltose[Title/Abstract])) OR (monofer[Title/Abstract])) OR (monoferric[Title/Abstract])) OR (diafer[Title/Abstract])) OR (iron isomaltoside[Title/Abstract])) OR (cosmofer[Title/Abstract])) OR (INFeD[Title/Abstract])) OR (iron[Title/Abstract])<br>AND<br>((anemia[MeSH Terms]) OR (anemia[Title/Abstract])) OR (anaemia[Title/Abstract])
EMBASE	'peritoneal dialysis'/exp OR 'home dialysis'/exp OR (('hemodialysis'/de OR 'artificial kidney'/exp OR hemodialys*:ab,ti,kw OR haemodialys*:ab,ti,kw OR 'hemo-dialys*':ab,ti,kw OR 'haemo-dialys*':ab,ti,kw OR 'renal dialys*':ab,ti,kw OR 'artificial kidney*':ab,ti,kw) AND (home:ab,ti,kw OR homebased:ab,ti,kw)) OR 'peritoneal dialys*':ab,ti,kw<br>AND<br>('iron'/exp) OR ('ferrous sulfate':ti,ab) OR ('ferrous gluconate':ti,ab) OR ('losferron':ti,ab) OR ('ferrous fumarate':ti,ab) OR ('ferrous chloride':ti,ab) OR ('iron dextran':ti,ab) OR ('ferric gluconate':ti,ab) OR ('iron sucrose':ti,ab) OR ('ferric saccharate':ti,ab) OR ('iron saccharate':ti,ab) OR ('venofer':ti,ab) OR ('ferric carboxymaltose':ti,ab) OR ('ferinject':ti,ab) OR ('ferric derisomaltose':ti,ab) OR ('monofer':ti,ab) OR ('monoferric':ti,ab) OR ('diafer':ti,ab) OR ('iron isomaltoside':ti,ab) OR ('cosmofer':ti,ab) OR ('INFeD':ti,ab) OR ('iron':ti,ab)<br>AND<br>('anemia'/exp) OR ('anaemia':ti,ab) OR ('anaemia':ti,ab)

4. Newcastle-Ottawa Scales for cohort and cross-sectional studies

NEWCASTLE - OTTAWA QUALITY ASSESSMENT SCALE
COHORT STUDIES

Note: A study can be awarded a maximum of one star for each numbered item within the Selection and Outcome categories. A maximum of two stars can be given for Comparability

Selection

1. Representativeness of the exposed cohort

a) truly representative of the average dialysis patient in the community ★
b) somewhat representative of the average dialysis patient in the community ★
c) selected group of users (eg nurses, volunteers)
d) no description of the derivation of the cohort

2. Selection of the non-exposed cohort

a) drawn from the same community as the exposed cohort ★
b) drawn from a different source
c) no description of the derivation of the non-exposed cohort

3. Ascertainment of exposure

a) secure record (eg surgical records) ★
b) structured interview ★
c) written self-report
d) no description

4. Demonstration that outcome of interest was not present at start of study

a) yes ★
b) no

Comparability

1. Comparability of cohorts on the basis of the design or analysis

a) study controls for age ★
b) study controls for any additional relevant factors ★

Outcome

1. Assessment of outcome

a) independent blind assessment ★
b) record linkage ★
c) self-report
d) no description

2. Was follow-up long enough for outcomes to occur

a) yes (select an adequate follow up period for outcome of interest) ★
b) no

3. Adequacy of follow up of cohorts

a) complete follow up - all subjects accounted for ★

b) subjects lost to follow up unlikely to introduce bias - small number lost - > 80% follow up, or description provided of those lost ★
c) follow up rate < 60% and no description of those lost
d) no statement

NEWCASTLE - OTTAWA QUALITY ASSESSMENT SCALE
ADAPTED FOR CROSS-SECTIONAL STUDIES

Selection

1. Representativeness of the sample:
a) Truly representative of the average in the target population. ★ (all subjects or random sampling)
b) Somewhat representative of the average in the target population. ★ (non-random sampling)
c) Selected group of users.
d) No description of the sampling strategy.

2. Sample size:
a) Justified and satisfactory. ★
b) Not justified.

3. Non-respondents:
a) Comparability between respondents and non-respondents characteristics is established, and the response rate is satisfactory. ★
b) The response rate is unsatisfactory, or the comparability between respondents and non-respondents is unsatisfactory.
c) No description of the response rate or the characteristics of the responders and the non-responders.

4. Ascertainment of the exposure (risk factor):
a) Validated measurement tool. ★★
b) Non-validated measurement tool, but the tool is available or described. ★
c) No description of the measurement tool.

Comparability

1. The subjects in different outcome groups are comparable, based on the study design or analysis. Confounding factors are controlled.
a) The study controls for the most important factor (select one). ★
b) The study control for any additional factor. ★

Outcome

1. Assessment of the outcome:
a) Independent blind assessment. ★★
b) Record linkage. ★★
c) Self-report. ★
d) No description.

2. Statistical test:
a) The statistical test used to analyze the data is clearly described and appropriate, and the measurement of the association is presented, including confidence intervals and the probability level (p value). ★
b) The statistical test is not appropriate, not described or incomplete.

This scale has been adapted from the Newcastle-Ottawa Quality Assessment Scale for cohort studies to perform a quality assessment of cross-sectional studies for the systematic review, “Are Healthcare Workers’ Intentions to Vaccinate Related to their Knowledge, Beliefs and Attitudes? A Systematic Review” (25).

We have not selected one factor that is the most important for comparability, because the variables are not the same in each study. Thus, the principal factor should be identified for each study.

In our scale, we have specifically assigned one star for self-reported outcomes, because our study measures the intention to vaccinate. Two stars are given to the studies that assess the outcome with independent blind observers or with vaccination records, because these methods measure the practice of vaccination, which is the result of true intention.

5. Quality assessment per study using the Newcastle-Ottawa Scale

Author, year: Bae, 2015
Study design: Prospective cohort

Domain	Item	Reponse option	Comments	Stars
Selection	Representativeness of the exposed cohort	Truly representative of the average dialysis patient in the community		*
	Selection of the non exposed cohort	Drawn from the same community as the exposed cohort		*
	Ascertainment of exposure	Secure record (eg surgical records)		*
	Demonstration that outcome of interest was not present at start of study	Yes		*

Comparability	Comparability of cohorts on the basis of the design or analysis	Study controls for age and additional relevant factors	Included factors: age, gender diabetes mellitus, previous cardiovascular disease history, duration of dialysis, serum level of iron, ferritin, albumin, intact PTH, hsCRP, total cholesterol and singe-pool KT/V	**
Outcome	Assessment of outcome	Record linkage		*
	Was follow-up long enough for outcomes to occur	Yes	Five years of follow-up for mortality	*
	Adequacy of follow up of cohorts	Subjects lost to follow up unlikely to introduce bias - small number lost - > 80% follow up, or description provided of those lost		*

Author, year: Chavers, 2004
Study design: Retrospective cohort

Domain	Item	Reponse option	Comments	Stars
Selection	Representativeness of the exposed cohort	truly representative of the average dialysis patient in the community		*
	Selection of the non exposed cohort	drawn from the same community as the exposed cohort		*
	Ascertainment of exposure	secure record (eg surgical records)		*
	Demonstration that outcome of interest was not present at start of study	yes		*
Comparability	Comparability of cohorts on the basis of the design or analysis	study controls for age and additional relevant factors	Stratification for age, sex and race	**

Outcome	Assessment of outcome	record linkage		*
	Was follow-up long enough for outcomes to occur	yes	Five years of follow up for hemoglobin trends	*
	Adequacy of follow up of cohorts	follow up rate < 60% and no description of those lost		

Author, year: Coronel, 2003
Study design: Cross-sectional

Domain	Item	Reponse option	Comments	Stars
Selection	Representativeness of the sample	Somewhat representative of the average in the target population		*
	Sample size	Not justified		
	Non-respondents	Comparability between respondents and non-respondents characteristics is established, and the response rate is satisfactory		*
	Ascertainment of the exposure (risk factor)	Validated measurement tool		**
Comparability	Subject in the different outcome groups are comparable, based on the study design or analysis. Confounding factors are controlled	study does not control for relevant confounding factors	Included factors: albumin, Hb and i.v. iron	

Outcome	Assessment of outcome	Record linkage		**
	Statistical test	The statistical test is not appropriate, not described or incomplete	Unadjusted testing, did not include important confounding factors	

Author, year: Deger, 2013
Study design: Cross-sectional

Domain	Item	Reponse option	Comments	Stars
Selection	Representativeness of the sample	Somewhat representative of the average in the target population		*
	Sample size	Not justified		
	Non-respondents	Comparability between respondents and non-respondents characteristics is established, and the response rate is satisfactory		*
	Ascertainment of the exposure (risk factor)	Validated measurement tool		**
Comparability	Subject in the different outcome groups are comparable, based on the study design or analysis. Confounding factors are controlled	study controls for age and additional relevant factors	Included factors: age, gender, ferritin, serum Fe, iPTH, dialysis modality, iron therapy, phosphate binder therapy, active vitamin d therapy	**

Outcome	Assessment of outcome	Record linkage		**
	Statistical test	The statistical test is not appropriate, not described or incomplete	No confidence intervals were given	

Author, year: Evans, 2020
Study design: Retrospective cohort

Domain	Item	Reponse option	Comments	Stars
Selection	Representativeness of the exposed cohort	truly representative of the average dialysis patient in the community		*
	Selection of the non exposed cohort	drawn from the same community as the exposed cohort		*
	Ascertainment of exposure	secure record (eg surgical records)		*
	Demonstration that outcome of interest was not present at start of study	yes		*
Comparability	Comparability of cohorts on the basis of the design or analysis	study controls for age and additional relevant factors	Included factors: age, stage (for non-DD), sex, myocardial infarction, peripheral vascular disease, cerebrovascular disease, heart failure, prior diabetes, prior statin and angiotensin-converting enzyme	**

			inhibitor/angiotensin receptor blocker, Hb level and hs-CRP (categorized into <3, 3–10, 10–20 and >20).	
Outcome	Assessment of outcome	record linkage		*
	Was follow-up long enough for outcomes to occur	no	1 year for major adverse cardiac events	
	Adequacy of follow up of cohorts	no statement		

Author, year: Gao, 2023
Study design: Cross-sectional

Domain	Item	Reponse option	Comments	Stars
Selection	Representativeness of the sample	Somewhat representative of the average in the target population		*
	Sample size	Not justified		
	Non-respondents	Comparability between respondents and non-respondents characteristics is established, and the response rate is satisfactory		*
	Ascertainment of the exposure (risk factor)	Validated measurement tool		**
Comparability	Subject in the different outcome groups are comparable, based on the study design or analysis. Confounding factors are controlled	study controls for age and additional relevant factors	Included factors: age, sex and ESA use	**

Outcome	Assessment of outcome	Record linkage		**
	Statistical test	The statistical test is not appropriate, not described or incomplete	No confidence intervals were given	

Author, year: House, 1998
Study design: Retrospective cohort

Domain	Item	Reponse option	Comments	Stars
Selection	Representativeness of the exposed cohort	somewhat representative of the average dialysis patient in the community		*
	Selection of the non exposed cohort	drawn from the same community as the exposed cohort		*
	Ascertainment of exposure	secure record (eg surgical records)		*
	Demonstration that outcome of interest was not present at start of study	yes		*
Comparability	Comparability of cohorts on the basis of the design or analysis	study controls for age and additional relevant factors	Included factors: age, gender, albumin, iron defi- with estimates ranging from US$200 to US$400 per ciency, parathyroid hormone (PTH), underlying renal unit.	**

Outcome	Assessment of outcome	record linkage		*
	Was follow-up long enough for outcomes to occur	Yes	2 years of follow-up to assess receiving blood transfusion	*
	Adequacy of follow up of cohorts	complete follow up - all subjects accounted for	1 patients was excluded due to incomplete information	*

Author, year: Lim, 2019
Study design: Prospective cohort

Domain	Item	Reponse option	Comments	Stars
Selection	Representativeness of the exposed cohort	somewhat representative of the average dialysis patient in the community		*
	Selection of the non exposed cohort	drawn from the same community as the exposed cohort		*
	Ascertainment of exposure	secure record (eg surgical records)		*
	Demonstration that outcome of interest was not present at start of study	yes		*
Comparability	Comparability of cohorts on the basis of the design or analysis	study does not control for confounding factors	A stepwise multiple linear regression model is mentioned but confounding factors are not identified	

Outcome	Assessment of outcome	record linkage		*
	Was follow-up long enough for outcomes to occur	no	6 months of dialysis is relative short to assess differences between modalities concerning anemia parameters	
	Adequacy of follow up of cohorts	subjects lost to follow up unlikely to introduce bias - small number lost - > 80% follow up, or description provided of those lost		*

Author, year: Malyszko, 2009
Study design: Cross-sectional

Domain	Item	Reponse option	Comments	Stars
Selection	Representativeness of the sample	Somewhat representative of the average in the target population		*
	Sample size	Not justified		
	Non-respondents	Comparability between respondents and non-respondents characteristics is established, and the response rate is satisfactory		*
	Ascertainment of the exposure (risk factor)	Validated measurement tool		**
Comparability	Subject in the different outcome groups are comparable, based on the study design or analysis. Confounding factors are controlled	study does not control for relevant confounding factors	A stepwise multiple linear regression model is mentioned but confounding factors are not identified	

Outcome	Assessment of outcome	Record linkage		**
	Statistical test	The statistical test is not appropriate, not described or incomplete	No adjustment for important confounding factors	

Author, year: Matsumara, 2020
Study design: Cross-sectional

Domain	Item	Reponse option	Comments	Stars
Selection	Representativeness of the sample	Somewhat representative of the average in the target population		*
	Sample size	Not justified		
	Non-respondents	Comparability between respondents and non-respondents characteristics is established, and the response rate is satisfactory		*
	Ascertainment of the exposure (risk factor)	Non-validated measurement tool, but the tool is available or described		*
Comparability	Subject in the different outcome groups are comparable, based on the study design or analysis. Confounding factors are controlled	study does not control for important confounding factors	No adjustedment for confounding factors	

Outcome	Assessment of outcome	Record linkage		**
	Statistical test	The statistical test is not appropriate, not described or incomplete	No adjustment for important confounding factors	

Author, year: Niikura, 2019
Study design: Cross-sectional

	Item	Reponse option	Comments	Stars
Selection	Representativeness of the sample	Somewhat representative of the average in the target population		*
	Sample size	Not justified.		
	Non-respondents	Comparability between respondents and non-respondents characteristics is established, and the response rate is satisfactory		*
	Ascertainment of the exposure (risk factor)	Validated measurement tool		**
Comparability	Subject in the different outcome groups are comparable, based on the study design or analysis. Confounding factors are controlled	study does not control for important confounding factors	Adjustment based on forward stepwise addition of covariates. Important confounding factors not included	*

Outcome	Assessment of outcome	Record linkage		**
	Statistical test	The statistical test is not appropriate, not described or incomplete	Unadjusted testing, did not include important confounding factors	

Author, year: St. Peter, 2005
Study design: Retrospective cohort

	Item	Reponse option	Comments	Stars
Selection	Representativeness of the sample	Somewhat representative of the average in the target population		*
	Sample size	Not justified.		
	Non-respondents	Comparability between respondents and non-respondents characteristics is established, and the response rate is satisfactory		*
	Ascertainment of the exposure (risk factor)	Validated measurement tool		**
Comparability	Subject in the different outcome groups are comparable, based on the study design or analysis. Confounding factors are controlled	study does not control for important confounding factors	Adjustment based on forward stepwise addition of covariates. Important confounding factors not included	*

Outcome	Assessment of outcome	Record linkage		**
	Statistical test	The statistical test is not appropriate, not described or incomplete	Unadjusted testing, did not include important confounding factors	

Author, year: Wetmore, 2015
Study design: Retrospective cohort

	Item	Reponse option	Comments	Stars
Selection	Representativeness of the exposed cohort	truly representative of the average dialysis patient in the community		*
	Selection of the non exposed cohort	drawn from the same community as the exposed cohort		*
	Ascertainment of exposure	secure record (eg surgical records)		*
	Demonstration that outcome of interest was not present at start of study	yes		*
Comparability	Comparability of cohorts on the basis of the design or analysis	study controls for age and additional relevant factors	Included factors: age, sex, race, cause of ESRD, and dialysis duration	**

Outcome	Assessment of outcome	record linkage		*
	Was follow-up long enough for outcomes to occur	no	A quarter year per patient	
	Adequacy of follow up of cohorts	no statement		

Author, year: Zhou, 2012
Study design: Cross-sectional

	Item	Reponse option	Comments	Stars
Selection	Representativeness of the sample	Truly representative of the average in the target population.		*
	Sample size	Justified and satisfactory		*
	Non-respondents	Comparability between respondents and non-respondents characteristics is established, and the response rate is satisfactory		*
	Ascertainment of the exposure (risk factor)	Validated measurement tool		**
Comparability	Subject in the different outcome groups are comparable, based on the study design or analysis. Confounding factors are controlled	study does not control for important confounding factors	Included factors: Triglyceride, MDA, Dialysis vintage, IHD morbidity, ferritin, BMI	

Outcome	Assessment of outcome	Record linkage		**
	Statistical test	The statistical test is not appropriate, not described or incomplete	Univariate t-testing, did not include important confounding factors	

Author, year: Zitt, 2014
Study design: Prospective cohort

	Item	Reponse option	Comments	Stars
Selection	Representativeness of the exposed cohort	somewhat representative of the average dialysis patient in the community		*
	Selection of the non exposed cohort	drawn from the same community as the exposed cohort		*
	Ascertainment of exposure	secure record (eg surgical records)		*
	Demonstration that outcome of interest was not present at start of study	yes		*
Comparability	Comparability of cohorts on the basis of the design or analysis	study controls for age and additional relevant factors	Included factors: age, sex, time-dependent type of renal replacement therapy, diabetes, time-dependent C-reactive protein, albumin and hemoglobin	**

Outcome	Assessment of outcome	record linkage		*
	Was follow-up long enough for outcomes to occur	yes	Maximum of follow-up of 8 years for mortality (including all-cause, cardiovascular and sepsis)	*
	Adequacy of follow up of cohorts	follow up rate < 60% and no description of those lost	After eight years and no description of those lost	

6. Supplementary table prevalence of iron therapy

Author, Year	IV iron use (%)		Oral iron use (%)	
	HD	PD	HD	PD
Bae, 2015[34]	11.7	1.6	58.6	64
Chavers, 2004[29]	82.5	20.3	*NA*	*NA*
Coronel, 2003[37]	77	49	*NA*	*NA*
Deger, 2013[31]	*NA*	*NA*	*NA*	*NA*
Evans, 2020[33]	69	25	1	12
Gao, 2023[40]	26	*NA*	*NA*	92
House, 1998[41]	*NA*	*NA*	45.5-55.5	34.1
Lim, 2019[32]	42.9	17.5	97.6	96.5
Malyszko, 2009[39]	78	*NA*	*NA*	100
Matsumura, 2020[38]	33	*NA*	*NA*	21
Niikura, 2019[35]	25	*NA*	*NA*	25
St. Peter, 2005[28]	84.4	19.3	*NA*	*NA*
Wetmore, 2015[27]	74.5	36.5	*NA*	*NA*
Zhou, 2012[36]	27.4	2.5	*NA*	*NA*
Zitt, 2014[30]	*NA*	*NA*	*NA*	*NA*

NA: Not available

7. Supplementary table dose and frequency of iron therapy

Author, Year	Mean dose of IV iron administration per 30 days (mg)		Mean dose of oral iron administration per 30 days (mg)		Frequency of iron administration per 30 days	
	HD	PD	HD	PD	HD	PD
Bae, 2015[34]	*NA*	*NA*	*NA*	*NA*	*NA*	*NA*
Chavers, 2004[29]	*NA*	*NA*	*NA*	*NA*	*NA*	*NA*
Coronel, 2003[37]	250 - 750	62.5 - 250	*NA*	*NA*	4 - 12	1 - 4
Deger, 2013[31]	*NA*	*NA*	*NA*	*NA*	*NA*	*NA*
Evans, 2020[33]	*NA*	*NA*	*NA*	*NA*	*NA*	*NA*
Gao, 2023[40]	*NA*	*NA*	*NA*	*NA*	*NA*	*NA*
House, 1998[41]	*NA*	*NA*	*NA*	*NA*	*NA*	*NA*
Lim, 2019[32]	108	*65*	*NA*	*NA*	*NA*	*NA*
Malyszko, 2009[39]	*NA*	*NA*	*NA*	*NA*	*NA*	*NA*
Matsumura, 2020[38]	160	*NA*	*NA*	3000	*NA*	*NA*
Niikura, 2019[35]	*NA*	*NA*	*NA*	*NA*	*NA*	*NA*
St. Peter, 2005[28]	*NA*	*NA*	*NA*	*NA*	*NA*	*NA*
Wetmore, 2015[27]*	246 - 307	127 - 151	*NA*	*NA*	2.9 - 3.4	0.8
Zhou, 2012[36]	*NA*	*NA*	*NA*	*NA*	*NA*	*NA*
Zitt, 2014[30]*	*209*	*209*	*1455*	*1455*	4	*4*

HD: hemodialysis, PD: peritoneal dialysis, IV: intravenous, NA: Not available
*Frequency only described for patients receiving intravenous treatment

8. Supplementary table anemia and iron serum markers

Author, Year	Mean haemoglobin (g/dL)		Mean ferritin (ng/ml)		Mean TSAT (%)	
	HD	PD	HD	PD	HD	PD
Bae, 2015[34]	10.7 ± 1.2	10.6 ± 1.5	292 ± 310	287 ± 323	32 ± 16	31 ± 15
Chavers, 2004[29]	*NA*	*NA*	*NA*	*NA*	*NA*	*NA*
Coronel, 2003[37]	11.6 ± 1.3	11.4 ± 1.4	338 ± 167	218 ± 214	23 ± 8	26 ± 11
Deger, 2013[31]	10.7 (8–14.5)*	10.6 (7.4–17.1)*	430 (34–1584)*	207 (41–1990)*	32 ± 16	31 ± 13
Evans, 2020[33]	11.3 ± 1.32	11.6 ± 1.39	*NA*	*NA*	*NA*	*NA*
Gao, 2023[40]	10.2 ± 2.2	9.6 ± 2.3	125 ± 84	116.9 ± 84.0	40 ± 13	39 ± 14
House, 1998[41]	10.5 ± 0.1	10.7 ± 0.1	259 ± 43	254 ± 57	29 ± 1	28 ± 1
Lim, 2019[32]	10.0 ± 1.0	10.6 ± 1.0	228 ± 124	260 ± 208	34 ± 13	38 ± 13
Malyszko, 2009[39]	11.9 ± 1.0	11.9 ± 1.6	288 ± 252	352 ± 313	*NA*	*NA*
Matsumura, 2020[38]	11.0 (10.5–11.7)*	11.0 (10.5–11.8)*	50 (29–71)*	119 (105–161)*	22 (16–28)*	37 (24–43)*
Niikura, 2019[35]	10.4 ± 1.5	10.3 ± 1.2	107 (45–178)*	134 (79–250)*	25 ± 10	34 ± 13
St. Peter, 2005[28]	*NA*	*NA*	*NA*	*NA*	*NA*	*NA*
Wetmore, 2015[27]	12.0 to 10.7	11.7 to 10.6	*NA*	*NA*	*NA*	*NA*
Zhou, 2012[36]	10.3 ± 2.0	10.7 ± 2.1	291 (118–590)*	142 (65–299)*	*NA*	*NA*
Zitt, 2014[30]	*NA*	*NA*	*NA*	*NA*	*NA*	*NA*

HD: hemodialysis, PD: peritoneal dialysis, TSAT: transferrin saturation, NA: Not available

* Value reported as median with interquartile range
